# The cellular slime mold *Fonticula alba* forms a dynamic, multicellular collective while feeding on bacteria

**DOI:** 10.1016/j.cub.2022.03.018

**Published:** 2022-05-09

**Authors:** Christopher Toret, Andrea Picco, Micaela Boiero-Sanders, Alphee Michelot, Marko Kaksonen

**Affiliations:** 1 Department of Biochemistry and National Centre of Competence in Research, Chemical Biology, University of Geneva, Geneva, Switzerland; 2 Aix Marseille University, CNRS, IBDM, Turing Centre for Living Systems, Marseille, France

**Keywords:** slime mold, amoeba, multicellularity, opisthokonta, protist, collective invasion, bacterial death phase, evolutionary cell biology, emerging models

## Abstract

Multicellularity evolved in fungi and animals, or the opisthokonts, from their common amoeboflagellate ancestor but resulted in strikingly distinct cellular organizations. The origins of this multicellularity divergence are not known. The stark mechanistic differences that underlie the two groups and the lack of information about ancestral cellular organizations limits progress in this field. We discovered a new type of invasive multicellular behavior in *Fonticula alba*, a unique species in the opisthokont tree, which has a simple, bacteria-feeding sorocarpic amoeba lifestyle. This invasive multicellularity follows germination dependent on the bacterial culture state, after which amoebae coalesce to form dynamic collectives that invade virgin bacterial resources. This bacteria-dependent social behavior emerges from amoeba density and allows for rapid and directed invasion. The motile collectives have animal-like properties but also hyphal-like search and invasive behavior. These surprising findings enrich the diverse multicellularities present within the opisthokont lineage and offer a new perspective on fungal origins.

## Highlights

• Unexpected bacterial-state-dependent culture conditions for *Fonticula alba*
• A multicellular invasion of bacterial food resources that is distinct from fruiting
• A leader-led invasive collectivity that is an emergent property
• Insights into the origins of invasive hyphal and fruiting multicellularity in dikarya

## Teaser

Toret et al. delve into the biology of the *Fonticula alba*, a unique cellular slime mold that is sister to fungi. They discover a cryptic, emergent, and invasive multicellular organization with parallels to invasive hyphae. This study sheds light on the multicellular organizations that occur within the fungi-animal divergence.

## Introduction

The crucial innovation from single cells to multicellular states has occurred over two dozen times in the evolution of eukaryotes and is central to the diversity of life.^1^^,^^2^ In its simplest form, multicellularity can be aggregates, sheets, or filaments of cells.^1^ A more complex multicellularity combining these arrangements into tissues has evolved more rarely in animals, fungi, and a few other lineages.^3^^,^^4^ Despite sharing a common ancestor, the basic multicellular mechanisms (cell-cell adhesion, cell-extracellular matrix [ECM] adhesion, or syncytia) of fungi and animals have diverged such that early morphological classifications grouped fungi with plants. These stark differences have made understanding multicellularity origins in the fungi/animal clade of opisthokonta challenging.

**Figure 1:**
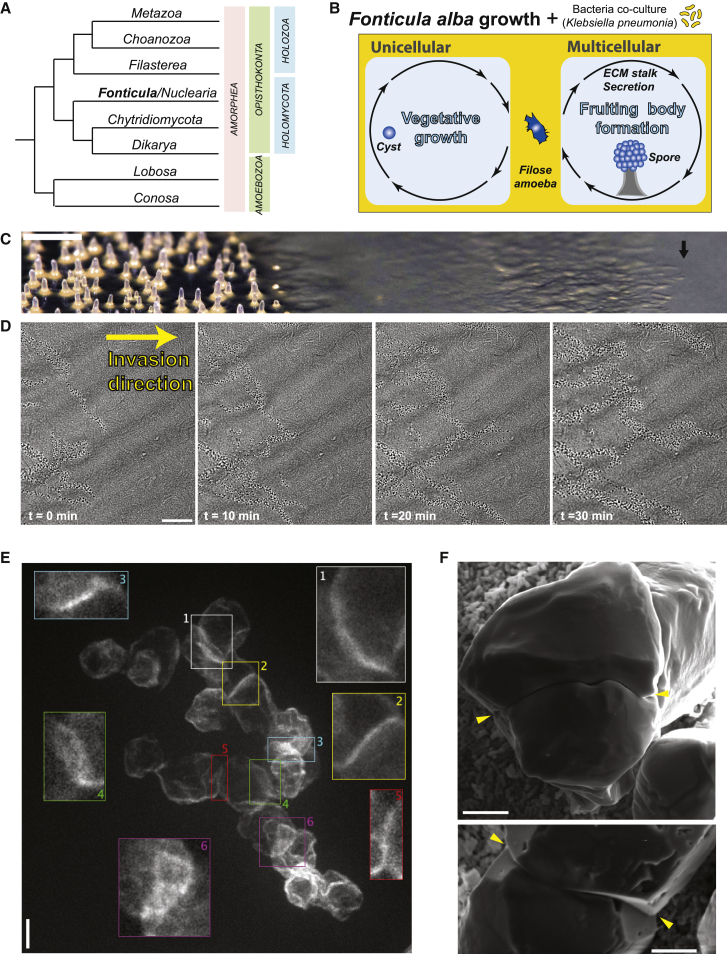
*F. alba* invades virgin bacterial sources collectively (A) Tree approximating *F. alb*a location in eukaryotic evolution (for a more detailed tree, see Galindo et al.^18^ and Brown et al.^19^). (B) Schematic of the known life cycle of *F. alba*. (C) Macro photograph of an *F. alba* colony radius growing on a plate; original sorocarp was placed left, and invasion into the bacterial resource is rightward (arrow marks the colony front). Scale bars, 1 mm. (D) Montage cell organization at the invasion front over time. Scale bars, 100 μm. (E) Maximal intensity z stack of a confocal section of a phalloidin-stained collective at the colony front. Colored boxes show enlarged single planes of individual cell-cell contacts of corresponding colored regions. Scale bars, 5 μm. (F) SEM region of two cell-cell interfaces (yellow arrowheads) present at the colony front. Scale bars, 5 μm. See also Figures S1 and S2, and Videos S1 and S2.

Advances in underexplored opisthokont lineages, such as filasterea, ichthyosporea, and choanoflagellates, have provided powerful insights into these questions in the holozoan branch.^5^, ^6^, ^7^ Animal cells share many hallmarks present in the amoebozoa outgroup, such as cell motility, phagocytosis, and epithelia formation, that offer insight into holozoan origins (Figure 1A).^8^^,^^9^ However, the fungal branch of opisthokonta has undergone a drastic transformation from an amoeboflagellate to dikarya, which contains the majority of known fungal species. Several features arose on the transitional path to dikarya. These include loss of cell motility and phagocytic processes, enclosure in a chitin-based cell wall,^10^ loss of cilia,^11^ saprophytic or parasitic transition,^12^^,^^13^ syncytial organization of cells within a hydrostatic system,^14^ an altered cytokinesis—which flowed through a syncytia-to-septation bottleneck—^15^^,^^16^ and two multicellularities that are of independent origins—a simple one for hyphal growth and a complex one for spore forming fruiting bodies.^17^ The order of events that gave rise to this remarkable evolutionary path remain unclear. Chytrids and other primitive fungi retain single cellular flagellate and amoeboid states, and emerging evidence suggests that rhizoid organizations common in chytrids may not predate hyphal arrangements.^18^^,^^17^

*Fonticula alba* is a curious social amoeba with a unique multicellularity within the opisthokont group (Figure 1A). Sequence analysis placed the organism in a group with nucleariids, which is sister to fungi.^19^ *F. alba* is a sorocarpic amoeba, which grows as single cells that aggregate together to create spore-filled fruiting bodies or sorocarps on a secreted volcano-like stalk of ECM. This cellular slime mold life cycle is similar to dictyostelids; however, the mechanism of sorogenesis and the multicellularity are distinct.^20^ Given its evolutionary position and unique biology and organization, *F. alba* has great potential to contribute to our understanding of opisthokont origins and divergence. However, harnessing this inadequately studied organism for investigation requires optimization of basic growth protocols.

## Results

### *F. alba* invades mature bacterial cultures with a multicellular organization

*F. alba* is a non-axenic social amoeba that requires co-culture with its bacterial food source.^20^^,^^21^ Previously, *Klebsiella pneumonia*, a common environmental and fecal bacteria, was defined as optimal for growth and fruiting, but *F. alba* can feed on other species.^20^ *F. alba* requires ∼1 week to produce fruiting bodies at 25°C, and American Type Culture Collection (ATCC) stocks contain *K. pneumonia* in co-culture. An isogenic *F. alba* was isolated from a single spore and maintained with a non-pathogenic *K. pneumonia* strain (KpGe) for this study. Growth of either a co-culture of *F. alba*-bacteria on a plate or a single *F. alba* sorocarp placed on a freshly seeded *K. pneumonia* bacterial lawn both took 5–7 days to produce fruiting bodies with a ∼3-day lag before *F. alba* activity was detectable (Figures S1A and S1B). However, when spores or cysts were placed on bacterial lawns that were 3–7 days old and incubated at 25°C, they reanimated (<24 h) and amoebae radiated outward and invaded the bacterial lawn to cover the entire plate (Figure S1B). During this invasion phase, a slight film radiated outward from the site of spore placement after 1 day (Figure S1B [dotted line]). A similar film formed on freshly plated bacteria but with a 3-day delay (Figure S1B [dotted line]). Partial depletion or thinning of the bacteria could be observed posterior to the invasion front and indicated a separate feeding front (Figure S1C [solid line]). Posterior to the feeding front, a fruiting zone occurs with the appearance of sorocarps. (Figure S1C [dashed line]).

The 3-day lag in activity on fresh bacteria was unexpected as the bacterial mat is already very dense after 24 h. Similar growth kinetics were observed in liquid culture of bacteria (further discussed later). To better understand *K. pneumonia* culture states in these growing conditions, we generated a bacteria growth curve and determined which time points allowed for *F. alba* amoeba activity by microscopy. *K. pneumonia* entered classical phases of bacteria growth over a 7-day period (Figure S1D).^22^ Over the time course, aliquots were analyzed for the presence of trophic amoebae 12 h after the addition of a sorocarp (Figure S1D [micrograph inset]). *F. alba* became active in bacterial cultures that were ∼3 days old or older. This time period of 3 days correlated with the end of the early stationary phase and the onset of the death phase, which corresponds to the time when bacterial death begins to exceed bacterial growth and is indicated by a decrease in colony-forming units (CFUs), while the dead cells continue to contribute to the optical density of the culture (Figure S1D). These results suggest that *F. alba* spore germination is dependent on bacterial culture age. The revised growth conditions allow for a 24 h growth period and are a marked improvement from the original week timescale.

Upon closer inspection of the border between the film front and the pristine bacterial lawn, an uneven edge was visible (Figure 1C [black arrow]). This colony front spread outward at a continuous rate followed by fruiting waves until the entire plate was covered. The uneven colony edge was visualized under the microscope, which revealed that the amoebae were organized into striking elongated, motile collectives that extended outward from the colony center (Figure 1D; Video S1, each black dot corresponds to a contractile vacuole, and approximates one cell). These collectives were dynamic and formed branches that fused and separated with great flexibility as they flowed in an outward direction into the virgin bacterial lawn (Figures 1D and S2; Video S1). Cell collectives migrated in close proximity to one another. Occasionally, collective units crossed over one another and maintained their direction of migration, suggesting a distinct identity and collective cohesion (Figure S2; Video S1). The collectives often migrated across the path of an earlier collective without following this prior path. This suggests that the collectives are not following any local cues or tracks in the bacterial lawn (Video S2). This collective migration appears to be used primarily for the invasion of the bacteria.

Video S1. Organization of the *F. alba* colony front, related to Figure 1 *F. alba* colony front on a *K. pneumonia* surface. Images captured at a 5 s interval (15 fps). Scale bars, 100 μm.

Video S2. Colony front dynamics over 2 h, related to Figure 1 A *F. alba* invasive collective *K. pneumonia* surface. Images captured at a 1 min interval over 2 h (15 fps). Scale bars, 100 μm.

Cell-cell contacts were examined in these collective invasions to better define group organization. Colony fronts were fixed and stained with fluorescent phalloidin to label the actin cytoskeleton. Cells had a predominant cortical actin network at the cell cortex (Figure 1E). Collectively organized cells were not arranged in flat sheets, and cell-cell contact regions appeared in three dimensions (Figure 1E). Regions of cell-cell contact had an actin enrichment along cell-cell borders (Figure 1E). The cell collectives were next analyzed by scanning electron microscopy (SEM). The cells had well-defined regions of direct contact that followed the cell contours of their neighbor, which suggests a direct cell-cell contact (Figure 1F). Approximately 25 cell-cell contacts were imaged; however, consistent with phalloidin staining, few were optimally oriented for the visualization of entire seams between cells by SEM, which further supports that cell organization in collectives is three-dimensional. Together, these results show that despite the fluid nature of invasive collectives, cell-cell interactions are organized and well-defined.

### A leader-follower organization occurs in invasive collectives

**Figure 2:**
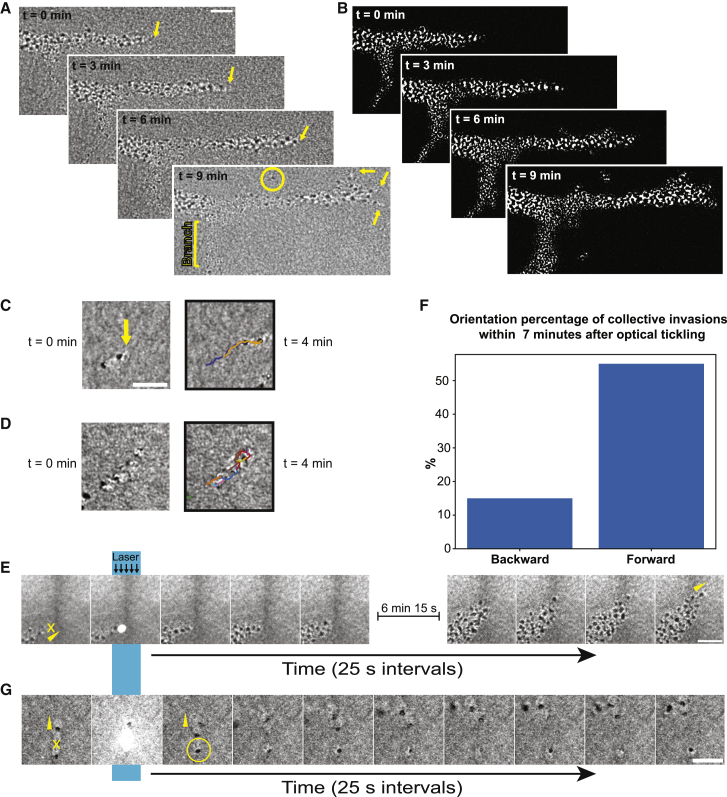
Invasive collectives use a leader-follower organization (A) Brightfield images of a single invasive collective at the front over time. The yellow circle indicates an individual cell escaping the collective and yellow arrows indicate single cell tips. (B) Median filter of images shown in (A) to digitally enhance contrast. (C) Image of two cells migrating together over time with a leader cell (yellow arrow). Right panel shows temporal trajectories overlaid on the last capture. (D) Montage of a collective over time which lacks a leader. Right panel shows temporal trajectories overlaid on the last capture. (E) Montage of video of individual invasive collectives upon optical tickling (marked by blue bar). Yellow arrowheads point in the direction of travel of the cells at the start and end of the video. Yellow “X” indicates the site of photobleach. (F) Quantification of post-tickling directions (n = 20). (G) Montage of video of individual invasive collectives upon optical tickling. Yellow circle indicates separated follower cell. All scale bars, 25 μm. See also Videos S3 and S4.

The *F. alba* collectives consisted of multiple cells linked together (Figures 2A and 2B; Video S3). Individual cells freely joined and left collectives, suggesting the cell-cell linkages are transient (Figures 2A [yellow circle] and 2B; Video S3A). Every collective that progressed forward had one cell leading it. When a branch formed, it started with the appearance of a new single cell leader at the side of an existing collective (Figures 2A [yellow arrows] and 2B; Video S3A). Collectives often consist of many cells but can form from just two cells that hook up head-to-tail, with one cell leading (Figure 2C [yellow arrow indicates leader]; Video S3B). Collectives that lacked a clear leader had cells that migrated within the collective but without resulting in productive movement of the whole collective (Figure 2D; Video S3C). These findings suggest a leader-follower organization.^23^

Video S3. Cell organization of collective invasions, related to Figure 2 (A–C) (A) A multi-cell *F. alba* invasive collective. (B) A two-cell collective. (C) A leaderless collective. All on a *K. pneumonia* surface. Images captured at a 5 s interval (15 fps). Scale bars, 25 μm.

To assay leader-follower dynamics, we exploited our observation that *F. alba* motility was sensitive to intense light. Focused pulses of a laser light (488 nm) resulted in temporary pauses (∼5 s) in cell migration. We used this behavior to optically “tickle,” or pause, single cells within a collective to understand the principles within the collective that drive the behavior. First, leader cells were tickled (Figure 2E; Video S4). Following a pause, collective cells reorganized into a cycling leaderless collective similar to Figure 2D that stopped progression. This state persisted until a new leader was promoted; however, the direction of travel was not always maintained. The time to promote a new leader appeared random, and some collectives (<40%) did not promote a leader within the 7 min imaging time. Quantification of 20 imaged collectives in which the leader was tickled revealed a bias in the orientation of the new leader toward the original orientation, outward from the colony center. However, the cells could also orient in the opposite direction (Figure 2F). These results indicate that leaders are necessary for directional progression. However, the global direction, outward from the colony center, may be controlled by more extrinsic factors or collective elements. Optical tickling of rearward cells revealed that when paused, the leader cell continued onward without delay (Figure 2G; Video S4) and indicates that leader cells are not responsive to followers. Together, these results indicate a dynamic leader-follower organization, which can be rapidly remodeled at the colony front during invasion.

Video S4. Optical tickling of collective invasions, related to Figure 2 Optical tickling of individual *F. alba* cells in collective on a *K. pneumonia* surface. Each panel shows a different independent collective (15 fps). Scale bars, 25 μm.

### Collective invasion is distinct from fruit formation

**Figure 3:**
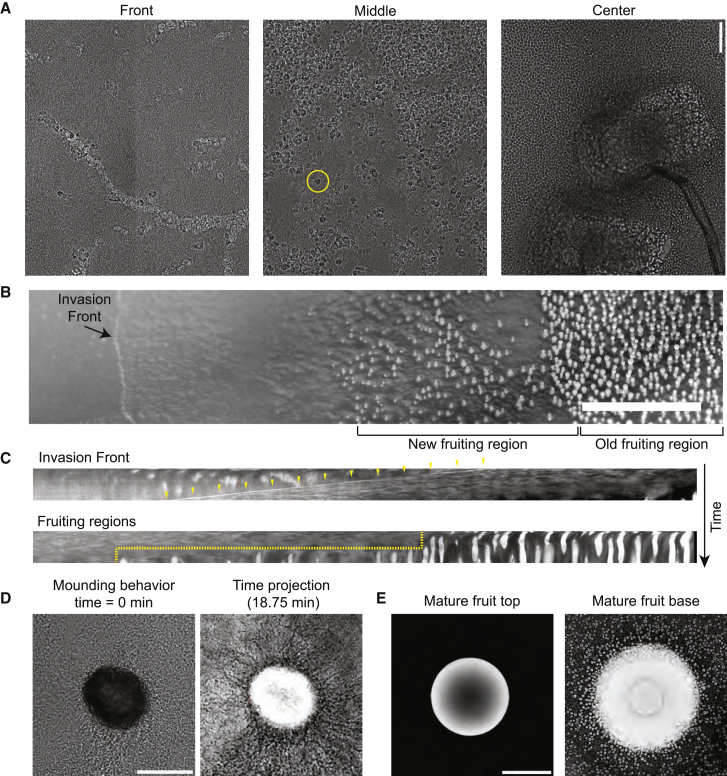
*F. alba* fruiting and invasive collectives are distinct (A) Three cropped regions from a radial section of an *F. alba* colony to show organization. Yellow circle is around one cyst. Scale bars, 50 μm. (B) Single plane from a ∼24-h macro photography video of an advancing invasion front and fruiting region. Scale bars, 5 mm. (C) Kymographs of invasion front (top) and fruiting front (bottom) from Video S3. Yellow arrowheads highlight the invasion front location over time. Dashed line shows the relative position of the fruiting front over time. (D) Brightfield image of an emerging fruit and surrounding cells (left). Inverted time projection of mound structure formation over 18.75 min (right). Scale bars, 100 μm. (E) Inverted brightfield images of a mature sorocarp, focused on the tip of the sorus (left) and base (right). Scale bars, 100 μm. See also Videos S5, S6, and S7.

We imaged the cell organization across a colony area extending from the invasion front to the fruiting regions to observe the relationship between invasive and fruiting collective behaviors (Figure 3A; Video S5). This approach approximates a time sampling as invasion regions mature to fruited regions. At the invasion front, cells were organized into outwardly directed linear collectives (Figure 3A [left panel]). More posterior to that, the collectives became progressively shorter and more randomly oriented until individual cells appeared and invasive collectives were no longer detectable (Figure 3A [middle panel]; Video S5). At this point, the cells amplified to a high density. Cysts were observed here, which are visible by the presence of an optically bright halo surrounding cells and darker interiors (for example, the yellow circle in Figure 3A), and suggest that many cells enter quiescent states in this dense intermediate region (Figure 3A; Video S5). After this point, cells appeared to re-enter an amoebic stage (Video S5) and fruit stalks emerged within the colony (Figure 3A [right panel]; Video S5). These results show that the collective activities of invasion and the fruit formation are distinct from one another. This organization is different from that observed in dictyostelids, where the starvation-induced collective streaming is the first step in the sorogenesis developmental pathway and the migration is directed toward the site where the fruiting body will form.^24^

Video S5. Panorama across a colony, front to center, related to Figure 3 A panorama display with Ken Burns effect created with iVideo of an imaged radial section of a *F. alba* colony on a *K. pneumonia* surface corresponding to Figure 2A.

*F. alba* colony growth was imaged over 24 h in invasive growth conditions (Figure 3B; Video S6). This analysis revealed that the invasion front advances at a continual rate of ∼1.25 cm/day (Figure 3C). However, fruiting occurs in a stepwise pattern, which is more consistent with a daily circadian cycle (Figure 3C). Four independent colonies of *F. alba* on aged bacterial plates all produced radial waves of fruit during the morning and throughout the day of the ∼24th, 48th, and 72nd h cycle of growth. The different rates of invasion and fruiting emphasize the distinctness of these two collective behaviors.

Video S6. Macrophotography timelapse of a colony, related to Figure 3 A macrophotography timelapse of a *F. alba* invasive front and fruiting front on a *K. pneumonia* surface. Images captured at a 10 min interval (15 fps). Scale bars, 5 mm.

Previously, *F. alba* was proposed to stream together prior to fruiting, like other social amoebae.^20^ Mound formation occurs when cells are hyper dense (Figure 3A; Video S5). Long linear collectives similar to the invasion front did not appear to form during the process (Figure 3D [left panel]; Video S7). Individual cells could be observed moving into the mound, but the high cell density made it difficult to determine if a few cells may still collectively migrate during this process. These observations suggest that the underlying collective behavior that drives sorogenesis is likely different from streaming during collective invasion. A time projection analysis revealed the presence of radial tracks into the mound (Figure 3D [right panel]), which suggests that some organization exists during mound formation. Many cells did not enter fruiting bodies but formed cysts at the base, which suggests that either sorocarp formation or encystation are optional fates for *F. alba* cells (Figure 3E). These observations demonstrate that two different multicellularities were built on top of a unicellular amoeba-cyst transition and reinforce that the multicellular mechanisms of *F. alba* mound and fruit formation are distinct from other social amoebae and from *F. alba* invasive collectives.

Video S7. Mound behavior prior to sorogenesis, related to Figure 3 *F. alba* mound formation on *K. pneumonia* surface. Images captured at a 5 s interval (15 fps). Scale bars, 25 μm.

### Collective invasion facilitates directed migration of amoebae

**Figure 4:**
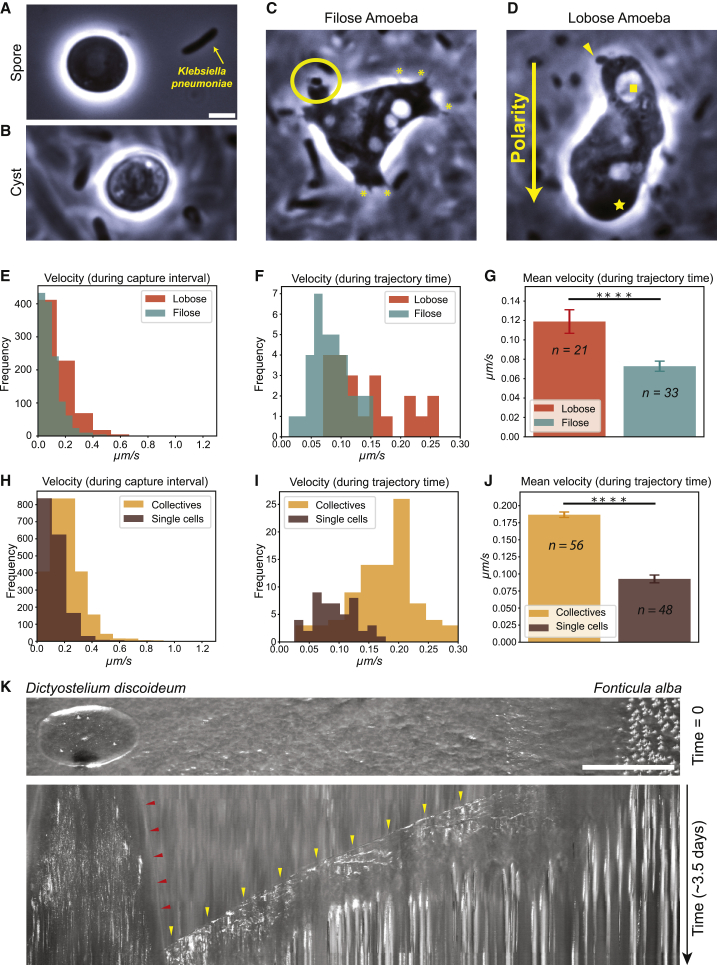
*F. alba* collective behavior favors invasion migration (A) Phase contrast image of spore immediately after placing a sorocarp in fresh media. (B) Phase contrast image of a cyst 24 h after inoculation of fresh media. (C and D) Phase contrast images of amoeba 24 h after inoculation into a 3-day-old culture of *K. pneumonia*. (A–D) Scale bars, 1 μm. (E) The velocity of lobose and filose amoebae during the exposure time interval (5 s). (F) The velocity of lobose and filose amoebae during the trajectory time, which is the time interval spanning the trajectory lengths. (G) Mean velocities of lobose and filose amoebae. p < 0.0001. (H) The velocity of single cells and collectives during the exposure time intervals (5 s). (I) The velocity of single cells and collectives during the trajectory time. (J) Mean velocities of single cells and collectives. p < 0.0001. (K) (Top panel: still image showing t = 0 of *D. discoideum* and *F. alba colonies* from Video S9. Scale bars, 5 mm. Bottom panel: kymograph from Video S9 with *D. discoideum* front advancement indicated by red arrowheads and *F. alba* front advancement by yellow arrowheads. (F, G, I, and J) The n values in (G and J) are the number of velocity data points during the trajectory time and also correspond to the respective histogram plots in (F and I). See also Videos S8 and S9.

Cysts and spores rapidly animated (∼5 h) in 3-day-aged KpGe liquid cultures (Figures 4C and 4D). Both a filose amoeba and a monopodial lobose amoeba form were readily detected in these updated growth conditions (Figures 4C and 4D). Cells transitioned between filose and lobose forms with relative ease. Filose amoebae had active filose spikes and membrane protrusions (Figure 4C [yellow asterisks]; Video S8A). The protrusions reached out into the media and engulfed bacteria from the surroundings (Figure 4C [yellow circle]; Video S8A). Filose amoebae were motile but changed their orientation frequently (Video S8A). Lobose amoebae were migratory and had a clear polarity with a single pseudopod indicating the direction of travel, and 95.3% (n = 107) had a single contractile vacuole located at the opposite end (Figure 4D [yellow star and yellow square, respectively]; Video S8B). These migratory lobose forms may not have been readily detected in previous conditions that do not favor collective invasion migrations. Curiously, lobose amoebae almost always had one or more bacteria in tow (Figure 4D [yellow triangle]; Video S8B). Bacteria engulfment was never observed in lobose form. The purpose of this behavior remains unknown, but a towing behavior may be important for collective invasion organizations. To quantify and better define these amoeboid behaviors, we tracked individual cells. We found that on glass coverslips lobose amoebae moved at a faster velocity than filose amoebae did during both a short time interval (the imaging interval of 5 s) and during the time spanning the entire trajectory length (Figures 4E and 4F). The mean velocity of lobose forms was greater than that of filose forms (Figure 4G). Speeds of *F. alba* amoebae were in the same range as reported for a single amoeba of the well-studied cellular slime mold *Dictyostelium discoideum*.^25^

Video S8. Amoeba states in *F. alba*, related to Figure 4 (A and B) (A) A *F. alba* filose amoeba (B) a lobose amoeba in liquid culture with *K. pneumonia*. Images captured at a 5 s interval (15 fps). Scale bars, 1 μm.

We also analyzed the motility of cells within an invasive collective migrating on bacterial mats on agar and the movement of individual cells at the colony front. During the image capture interval (5 s), single cells and cells in collectives had a similar speed (Figure 4H). However, by maintaining directed motility, collectively organized cells achieved much higher velocities over longer time intervals (Figures 4I and 4J). These results reveal a major advantage of collective organization for productive, directed travel into the untapped bacterial resource.

To illustrate the invasion advantage of *F. alba* collectives, a single *D. discoideum* and *F. alba* sorocarp were placed on an aged bacterial lawn and their expansion was imaged over ∼3.5 days (Figure 4K; Video S9). *F. alba* was able to cover the bacterial resource at a far greater rate than *D. discoideum* and produced vastly more fruiting bodies, highlighting the potential ecological advantage of collective invasion. On fresh bacteria, *D. discoideum* cultures would have a ∼3-day head start, and these differences may reflect ecological niches and different feeding strategies.

Video S9. Slime mold versus slime mold, related to Figure 4 A macrophotography timelapse of a *F. alba* and *D.discoideum* on a *K. pneumonia* surface. Images captured at a 10 min interval (15 fps). Scale bars, 5 mm.

### Collective invasion is cell density- and bacteria-dependent

**Figure 5:**
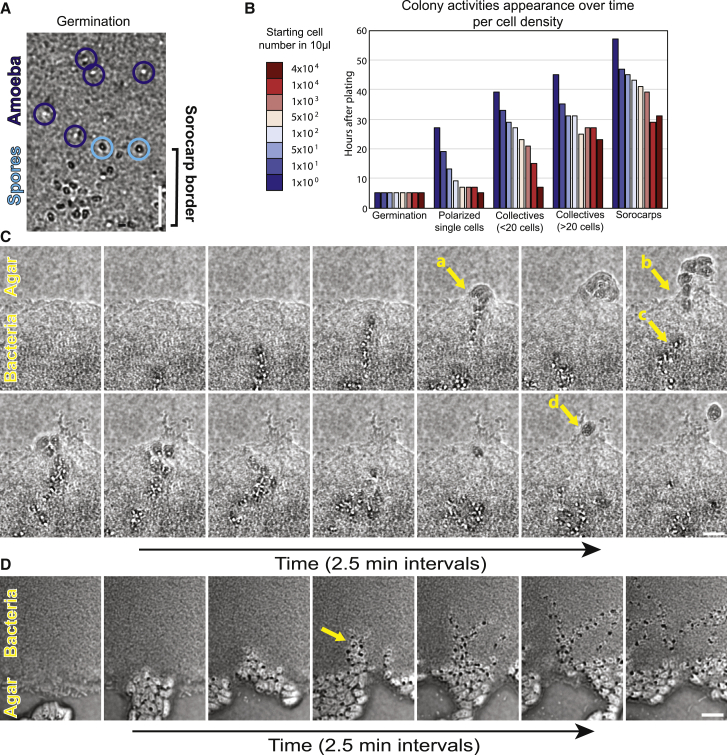
Invasive collectives are density- and bacteria-dependent (A) Single brightfield capture of sorocarp edges 5 h after placement on an aged *K. pneumonia* lawn. A single amoeba (purple) and spores (blue) are circled. (B) Plot of an *F. alba* colony featuring appearance over time in relation to starting spore density. (C) Video montage of an invasive collective as it encounters a bacteria colony-agar transition. (D) Video montage of individual *F. alba* cells on agar as they encounter an untouched bacterial colony. Scale bars, 25 μm. See also Videos S10 and S11.

Cells that emerge from a freshly seeded sorocarp after 4–5 h on an aged KpGe plate were imaged to identify when collective invasion was initiated. Single amoebae appeared along the edge of the sorocarp after germination. Cells lack a strong contractile vacuole at this stage and motility appears randomly oriented (Figure 5A; Video S10). These behaviors are consistent with filose amoebae, and cells are likely feeding in non-collective states at this early point after germination. These results suggest a transition from single cell states to the social invasion of mature colonies, but visualization of a distinct and direct change in behavior was inconclusive and is possibly an emergent property based on cell density. To test this possibility, colonies that initiated from different spore densities were microscopically examined over time and scored when landmark events occurred (Figure 5B). On these plates, germination was initiated in each condition after ∼5 h, which revealed that germination is independent of cell density (Figure 5B). Plates were then scored for an outwardly directed movement by the appearance of (1) individual polarized cells with distinct contractile vacuoles (lobose amoebae) and an orientation outward from the colony center; (2) small one-cell wide, head-to-tail collectives (under 20 cells); (3) complex collectives of over 20 cells; and (4) fruiting bodies. The invasive and collective properties all appeared over time in a density-dependent manner, with the cells at the highest concentration immediately progressing to collective invasion after germination (Figure 5B). These data suggest that collective invasion is an emergent property.^26^

Video S10. Spore germination, related to Figure 5 *F. alba* spore germination 5 h after placement on a *K. pneumonia* lawn. Images captured at a 5 s interval (15 fps). Scale bars, 25 μm.

The role of the bacteria in collective invasion was next analyzed. KpGe plates were spread unevenly to create bare patches in the bacterial lawn. Invasive collectives were visualized as they encountered these food deserts. Strikingly, cells in the collectives that encountered the bacteria-free agar underwent a rapid morphology change and flattened (Figure 5C [yellow arrow “a”]; Video S11A). Soon after entering the agar, the majority of cells reoriented and returned to the bacterial lawn (Figure 5C [yellow arrow “b”]). Cells that reentered the bacterial lawn appeared to redirect cells at the rear and prevent further outward movement (Figure 5C [yellow arrow “c”]). These data suggested that there are likely directional cues and potential communication between cells that remain to be understood. However, not all cells remained in the bacterial lawn and a few escapers ventured out into the agar (Figure 5C [yellow arrow “d”]). Cells migrating on an agar clearing remained in a flat morphology. On large clearings, cells eventually became cysts. Cells that encountered the new virgin bacterial feeding grounds reentered the bacterial lawn and promptly regained the collective head-to-tail morphology (Figure 5D [yellow arrow]; Video S11B). Taken together, these results demonstrate that collective invasive behavior is dependent on the immediate virgin bacterial environment.

Video S11. Bacterial dependence of collective invasion, related to Figure 5 (A) *F. alba* invasive collectives transitioning from a *K. pneumonia* lawn to an agar surface. (B) *F. alba* amoeba transitioning from an agar surface to collective behavior on a *K. pneumonia* lawn. Images captured at a 5 s interval (15 fps). Scale bars, 25 μm.

### Cytokinesis completion is inefficient in *F. alba*

**Figure 6:**
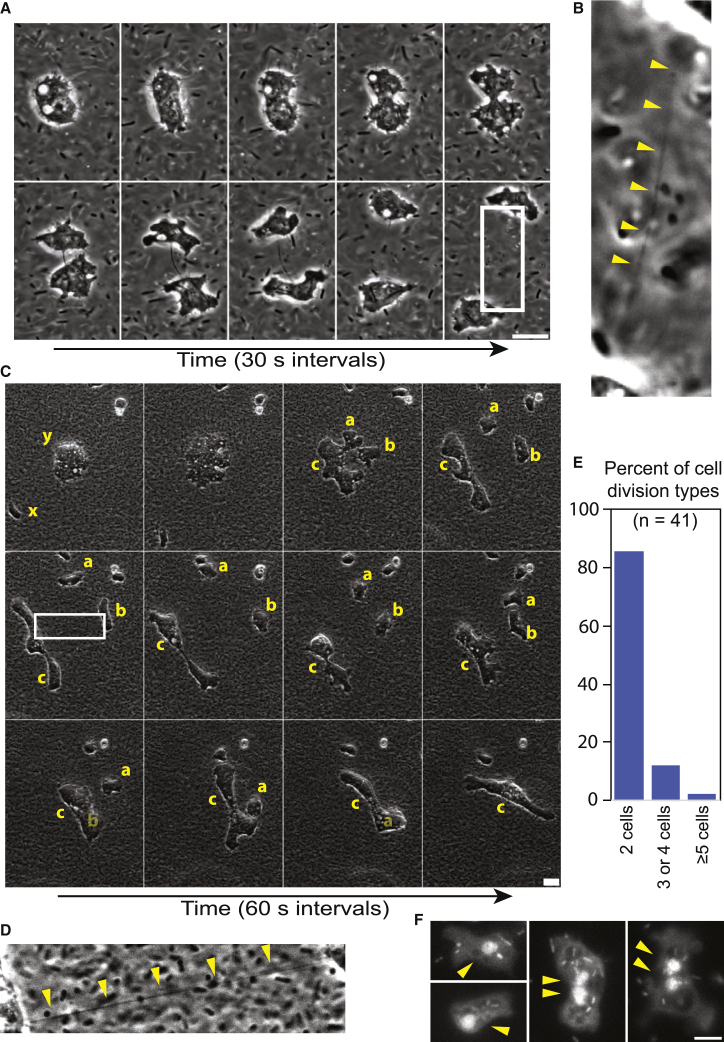
*F. alba* cells divide in aged bacterial cultures (A) Phase contrast image of cell division in a 24-h *F. alba* culture in aged bacteria. (B) Enlarged view of white-boxed region of (A). Yellow arrows indicate the membrane tube between cells. (C) Phase contrast image of a large cell division in a 24-h *F. alba* culture in aged bacteria. Yellow “x” indicates a small cell, yellow “y” indicates a large cell before division, and yellow “a,” b,” and “c” indicate daughter cells and are faded upon merging. (D) Enlarged view of white-boxed region of (C). Yellow arrows indicate the membrane tube. (E) Quantification of the percent of cell division events based on progeny from 10 fields of ∼50–100 cells imaged for 30 min. (F) Fixed cells stained with Hoescht. Yellow arrows indicate nuclei. (A, C, and E) Scale bars, (A and C) 10 μm, (E) 5 μm. See also Video S12.

In a spreading colony, most cellular amplification appears posterior to collective invasion (Figure 3A). The division of amoebae was analyzed in more detail in liquid cultures. The large majority of cells underwent a stereotypic division with a cell rounding up and dividing into two cells within ∼5–10 min (Figure 6A; Video S12A). At this rate displacement by cell division is orders of magnitude slower than collective invasion speeds, which is in agreement with collective migration being the predominant means of colony spread. During these mitotic analyses, chromosome condensates were never observed by phase microscopy, which favors a fungal-like closed mitosis for *F. alba* consistent with earlier EM observations.^21^ One key feature that we frequently observed was an extended cytokinetic bridge. During cytokinesis, the two cells separated and appeared to form and move independently, but a fine cytoplasmic tether persisted between the cells (Figure 6B [red triangles]; Video S12A). It was impossible to determine precisely how long tethers persisted, since their thickness decreased with increasing daughter cell separation and became hard to detect.

Video S12. Cell division in F. alba, related to Figure 6 (A) *F. alba* amoeba binary division. (B) *F. alba* abnormal division of a large amoeba. Cells grown in overnight liquid culture. Images captured at a 15 s interval (10 fps). Scale bars, 10 μm.

In addition, cells were sometimes found to divide into multiple progenies. This situation occurred only in large cells, which were ∼5%–10% of the cells in the culture (Figure 6C [x = normal size, y = large size]; Video S12B). Large cells, similar to small cells, rounded up prior to mitosis. However, more than two daughters were produced (Figure 6C [“a,” “b,” and “c”]; Video S12B). Daughters sometimes appeared to form normally and separate, but often well-formed cells were seen to retract or fuse back together (Figure 6C [“a” and “b”]; Video S12B). The retraction or merger of cells appeared to occur via cytokinetic tethers (Figure 6D). Mitotic events were scored for occurrence of binary versus multiple daughter cells, with ∼10 percent of mitotic events being non-binary, and rarely did events give rise to more than cells (Figure 6E). To image the nuclear states, cells were imaged with Hoescht DNA dye. Hoescht dye labeled both rod-shaped internalized bacteria and round nuclei (Figure 6F). Most cells contained one nucleus; however, larger cells often had two nuclei (Figure 6F). Together, these results are consistent with the initial account that a low percentage of *F. alba* cells appeared bi- and tri-nuclear.^20^ These data suggest that *F. alba* cells predominantly contain one nucleus but are tolerant of multinuclear states. Retraction via cytokinetic tethers is a plausible explanation for these multinuclear states, but it is necessary to develop nuclear imaging tools to visualize individual mitotic events in cells to be certain.

## Discussion

**Figure 7:**
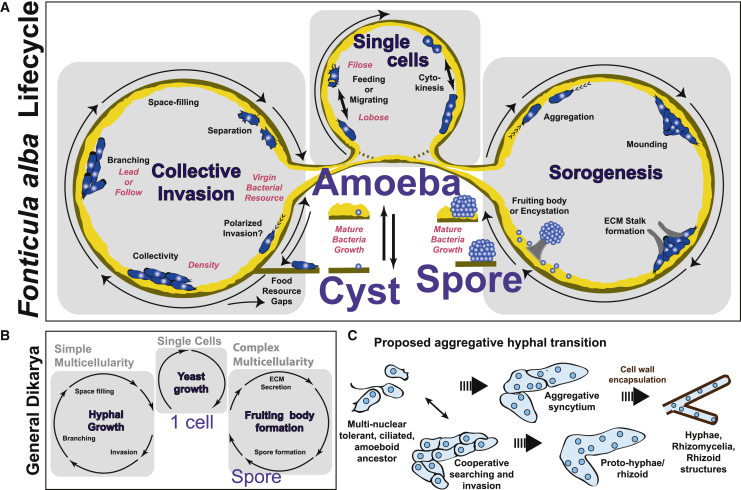
Life cycle schematics (A) Detailed *F. alba* life cycle schematic with *K. pneumonia* associations. (B) Life cycle of a stereotypic fungi in the dikarya clade. (C) Proposed aggregative route for the origin of fungal hyphae.

Our results redefine the life cycle of *F. alba* to include two optional social cycles, one for collective invasion and another for sorogenesis (Figure 7A). *F. alba* has an intimate tie to its bacterial food via culture-age-dependent germination and collective invasion. Germination requires bacterial cultures in which bacteria growth has ceased and death has begun.^22^ This phase of bacteria growth is poorly studied, and several factors may be sensed by *F. alba*, such as nutrient depletion, metabolite production, quorum sensing, lysed materials from cell death or other factors.^22^^,^^27^ A growth dependence on aged cultures may provide several benefits, such as a guaranteed large food reservoir, and vulnerable or weakened prey. Collective invasion only occurs when an untapped bacterial source is present and not when *F. alba is* grown in a homogenous mixed co-culture with bacteria when spreading or invasion are not necessary. At terminal stages of colony development, many *F. alba* cells do not enter fruiting bodies and remain as individual cysts in the substrate. This may serve as an environmental hedge strategy, creating both a means for dispersal (sorocarps) and a local reserve population (cysts) in case a food source is reinvigorated. These optional multicellular cycles are built on top of an ancient eukaryote amoeba-cyst transition.^28^ The identification of an entirely novel multicellular organization hidden in an unexpected link to bacterial culture age suggests that other emerging protist models may similarly hide unexpected discoveries. The related nucleariids, which do not form fruiting structures, may also exhibit food-related collective search and invasion behaviors yet to be discovered.^29^ Prokaryote-eukaryote relationships are an emerging field of metazoan development,^30^ and *F. alba* offers a new perspective on this association. The growth conditions identified in this study offer new strategies to discover other elusive and unculturable protists, which hold the potential to reshape our understanding of eukaryotic origins.

The two distinct *F. alba* multicellularities are significant for metazoan and fungal multicellularity divergence. The invasive collectivity is reminiscent of metazoan cell behavior and cancer states, which use leader and follower dynamics for invasion.^31^ *F. alba* cellular morphologies change on different substrates (bacteria versus agar). This behavior may be related to substrate influences observed in metazoan cell migration.^32^ It is unknown how the signaling and organizational pathways work for *F. alba’s* collective invasion. The nature of the link between *F. alba* cells in invasive collectives remains to be identified. Cells have defined actin-enriched cell-cell contacts, but the *F. alba* genome lacks any obvious cadherins, integrins, *Dictyostelium*-like cell adhesion molecules, or chitin and cellulose synthases, which constitute cell-cell adhesion systems in other clades.

Strikingly, the dual invasion and fruiting collectivity is reminiscent of fungal growth (Figure 7B). Specifically, the simple multicellularity of hyphae that search and invade food sources and the complex multicellularity that is built around ECM proteins and spore formation.^4^^,^^17^ In addition, the *F. alba* tolerates multinuclear states and has a weak cytokinesis (Figure 6). All three of these traits are expected to have arisen in the amoeba-to-fungi transition. Collective invasion demonstrates an alternative to a rhizoid- or neurite outgrowth-like origin for hyphae. We suggest a hypothesis where hyphae could have had a direct aggregative origin (Figure 7C). The last common ancestor of fungi and *F. alba* may have assembled amoeboid cells into head-to-tail arborized invasive collectives. Large syncytia-tolerant amoebae may have laid the foundation for multicellular networks, and cell wall encapsulation within early fungi would have been beneficial to buttress these large syncytial networks. Both metazoan focal adhesion machinery and the fungal Spitzenkörper machinery have ancestral ties,^33^ and further analysis of these pathways in *F. alba* may help resolve hyphal origins. Recent evidence suggests hyphal morphogenesis genes predate fungal multicellularity, and an aggregative origin to hyphae could explain this occurrence.^18^ Multinuclear states are common in many protists.^34^ Perhaps similar multinuclear and invasive evolutionary routes occurred in other protist branches such as myxomycetes and oomycetes.^35^^,^^36^ Fungal complex multicellularity mechanisms evolved independently at least 12 times. *F. alba* sorogenesis may represent a rudimentary concept of fungal complex multicellularity, being three-dimensional structures that are primarily based on ECM deposition, and may reflect a common holomycotan principle.^4^ Similarly, collective invasion and hyphal organization may be convergent strategies to serve an invasive function. The dependence of *F. alba* on dying bacterial cultures may also hint at the saprotrophic origins of fungi, but more work is needed to resolve the underlying mechanisms.

In addition to the single versus collective options, *F. alba* exhibits a number of choices during its life cycle. These include germination, whether to be a leader or follower (Figure 1), to be filose or lobose (Figure 4), to stay or migrate out of a bacterial lawn (Figure 5), and to be multinuclear or not (Figure 6). What regulates these behaviors remains unknown, and both intrinsic factors and extrinsic factors such as the environment (bacteria and/or circadian cycles) may be involved. The nature of these collective invasions raises questions about multicellularity. How long do the cells have to maintain a contact to be multicellular? How much adhesion is needed? Does there have to be a defined switch between unicellular and multicellular states or can it be a gradually emerging property? Multicellular states across biology likely exist on a gradient rather than a sharp cutoff. The transient nature of collective invasion suggests that it is at the weaker end of the multicellularity scale. *F. alba* reveals a novel single cell-to-multicellular transition in a unique branch of opisthokonta, and this makes it a powerful model organism to explore such evolutionary questions in development and cell biology.

## STAR★Methods

### Key resources table

**Table:** 

REAGENT or RESOURCE	SOURCE	IDENTIFIER
**Biological samples**		

*Fonticula alba*	ATCC	38817
*Dictyostelium discoideum*	Thierry Soldati lab, University of Geneva	AX2
*Klebseilla pneumonia*	https://doi.org/10.1016/j.micinf.2018.04.001	KpGe

**Chemicals, peptides and recombinant proteins**		

Yeast Extract	BD	212750
Bacto Peptone	BD	211677
Glucose	Sigma	G8270
Agar	BD	214010
K_2_HPO_4_	Applichem	122333.121
Formaldehyde (37-41%)	Fisher	10170052
Alexa488-Phalloidin	Invitrogen	A12379

**Deposited data**		

Tracking and speed quantification data	This study	https://github.com/apicco/Fonticula_tracking
Laser Tickling images for collective behavior quantifications	This study	https://doi.org/10.5281/zenodo.6303417

**Software and algorithms**		

ImageJ	Image analysis software	https://www.moleculardevices.com/systems/metamorph-research-imaging
VisiView	Microscopy Software	https://www.visitron.de/products/visiviewr-software.html
Metamorph	Microscopy Software	https://www.moleculardevices.com/systems/metamorph-research-imaging
Python 3.	Programming language	https://www.python.org/
Trajalign python library	Picco and Kaksonen, 2017^37^	https://github.com/apicco/trajectory_alignment/tree/fontiula_tracking
Scripts	This study	https://github.com/apicco/Fonticula_collective_invasion https://github.com/apicco/Fonticula_tracking

### Resource availability

#### Lead contact

Further information and requests for resources and reagents should be directed to and will be fulfilled by the lead contact, Marko Kaksonen (marko.kaksonen@unige.ch).

#### Materials availability

The clonal isolate of *F.alba*, Hän02, generated in this study is available upon request.

### Experimental model and subject details

#### Media, strains and culturing methods

*F. alba* growth media (YPPD) consisted of 5g/L yeast extract, 10g/L bacto-peptone, 10g/L glucose and 1g/L K_2_HPO_4_, pH 7.5 - 8.0 and 20g/L agar supplemented for plates. Media appears likely to support bacteria growth primarily, and the bacteria support *F. alba*. For bacteria-seeded plates a suspension of non-pathogenic *K. pneumonia* (KpGe)^38^ was spread on plates and allowed to dry (either rapidly in a tissue culture hood or slowly upright over 3 days in an incubator). The starting bacteria density was not critical beyond the need to create a lawn of bacteria in 24 hrs. Lower density suspensions that result in individual colonies prevent cells from reaching stationary phase as quickly and delay germination time. For liquid cultures bacteria was inoculated into liquid media (shaking). Both conditions incubated at 24°C standardly for 3 days or unless specified otherwise. Bacteria that were grown and aged at 37°C were not competent for *F. alba* growth at 24°C. KpGe-plates and cultures were stored at 4°C for up to 2 months without loss of activity. *F. alba* was only isolated once from the wild and no information exists whether or not sexess exist in this organism.

*D. discoideum* strain AX2(Ka) was obtained from the Soldati group (Univ. of Geneva). *F. alba* was obtained from ATCC (38817). *F. alba* is a non axenic strain and the ATCC resource stock exists as a co-culture with its food *K. pneumoniae* (Trevisian), a pathogenic strain. (Note: upon reanimation the stock contained a yeast-type fungal contaminant.) The associated bacteria was swapped with KpGe as described below. To isolate an isogenic line, individual sorocarps were selected on a dissection microscope with an 18 gauge needle and transferred to a 20g/L agar-only plate. A micro manipulator typical for yeast tetrad dissections ^39^ was used to isolate and move individual spores to marked locations. Spores were excised on agar slabs and inverted onto individual KpGe-plates. Isolates were frozen in 25% Glycerol and maintained in liquid nitrogen storage. All isogenic lines appeared identical in terms of growth, and an isolate named Hän02 was randomly chosen for all work in this study.

*F. alba* was passaged in the following manners. Primarily, one to ten sorocarps were selected using an 18 gauge needle on a dissection microscope and transferred to a KpGe- plate. Additionally, from a fruited plate both a sterile inoculating loopful of sorocarps and bacteria or an excised agar chunk inverted onto a different KpGe-plate could transfer the organism, but this method resulted in decreased fruiting efficiency over time for unknown reasons. A similar fruiting efficiency arose when cultures were maintained in liquid culture for long periods. However, these alternatives had no apparent loss in viability despite fewer fruit. Robust fruiting could be restored by selecting fruiting bodies for one or two generations. For co-culture methods, batch spore collection was done using a fully fruited 10 cm-dish. A pipet with sterile water was used to carefully dislodge and resuspend the *Fonticula-Klebseilla* mix. A second wash was pooled with the first to collect all spores and cysts, which was diluted (1/100) and spread on to a non-seeded media plate. The co-culture method is prone to contamination.

### Method details

#### *K. pneumonia* growth curve and *F. alba* activities

50 μl of an overnight KpGe culture in YPPD was inoculated into three independent culture flasks with 500 ml YPPD. For each time point over a 7 day period: Absorbance (600 nm) was measured, in addition, 10 fold serial dilutions were plated on YPPD plates to determine colony forming units. Lastly, one *F. alba* sorocarp was added to a 100 μl aliquot of each time point. After 12 hrs *F. alba-containing* aliquots were allowed to settle on a glass coverslip for 30 minutes and imaged by brightfield and scored for the presence of amoeba.

#### Bacteria association swap

Spores were collected in batch (as described above for co-culturing) and collected by low speed centrifugation (∼100 g) for 5 minutes and washed 4 times with sterile ddH_2_0. In the final wash spores and stalks have a snow globe-like effect with low turbidity. Cells were resuspended in *F. alba* growth media with 50 μg/ml kanamycin and incubated shaking at 24°C overnight to verify no bacteria growth. Cells were then washed 3 times in *F. alba* growth media and inoculated with KpGe and plated on an agar plate until fruit formation and cultured as described above.

#### Phalloidin staining

A *F. alba* sorocarp was placed on an aged-bacteria lawn and allowed to grow for ∼36 hrs until a clear invasion front was visible. The plate was then cooled to and maintained at 4°C. A 2% Low melting point agarose in 1x PBS solution at 30-37°C was gently overlaid over the plate surface until it covered the lawn surface and solidified rapidly at 4°C. In a minimal time, a region of the invasion front was excised with a scalpel and the agar-bacteria-fonticula-agarose sandwich was transferred to a 3.7% formaldehyde in 1X PBS bath for 1 hr at 4°C. The sandwich was then placed in 5% glycine 0.1% Trition X-100 1X PBS for 30 minutes. The sandwich separated and the agarose portion was placed, fonticula-bacteria side down in a drop of 1x PBS and maintained on a sheet of parafilm. The agarose slab was washed 4 times with 1x PBS by decanting and addition of new PBS. After the final wash, the agarose slab was left for 1 hr in a drop that was sufficient to cover the surface of 1x PBS containing 2U of Alexa Fluor 488 Phalloidin (Invitrogen). The sample was again washed 4 times by decanting and placed on a glass coverslip for immediate confocal imaging.

#### Mitotic quantification

10 fields of an overnight culture (∼50-100 cells/field) were imaged at 20x for 30 minutes. All mitotic events were counted and binned based on the number of progeny that appeared after division.

#### Nuclei labeling

An 100 μl drop from overnight *F.alba* liquid culture was allowed to settle on a coverglass for 20 minutes. The media was aspirated and replaced with 3.7% formaldehyde in 1X PBS for 6 minutes to fix. Cells were simultaneously quenched and permeablized in 100 μl of 5% glycine 2% trition X-100 1X PBS for 10 minutes. 100 μg/ml RNAse was added to the sample for an additional 10 minutes. (Nuclei penetration proved challenging and RNAse was effective at overcoming this issue for unknown reasons.) Coverglasses were washed 3 times with PBS and labeled with Hoescht (Santa Cruz Biotechnology, Inc) for 5 minutes. Coverglasses were then mounted in 80% glycerol and sealed with nail polish. All steps were done at room temperature.

#### Spore density growth assays

Spores were collected from a fully fruited plate and washed as described above. Spore densities were determined and adjusted with a hemocytometer. For each density three 10 μl spots were placed on a KpGe plate (in triplicate). Plates were visually scored under the microscope every 2 hrs for the appearance of amoeba, amoeba tracks outward from the inoculation site, small amoeba collectives (<20 cells), complex amoeba collectives (>20 cells) and fruiting bodies.

#### Scanning electron microscopy

A *F. alba* sorocarp was placed on an aged-bacteria lawn and allowed to grow for ∼36 hrs until a clear invasion front was visible. An agar slab that encompassed the entire *F. alba* colony was excised and gently placed into a glass dish. All solutions were slowly and gently added or removed at the base of the glass dish in a region away from the agar to minimize disruption of the agar slab surface. The agar slab was submerged in 2% osmium tetroxide, 1x PBS for 2 hrs and then washed 3 times in 1x PBS. Dehydration was then performed with a series of 1 hr washes (water, 30% ethanol, 50% ethanol, 70% ethanol, 95% ethanol and 100% ethanol). The following day the wash series was continued (100% acetone, 100% acetone, 2:1 (hexamethyldisilazane: acetone), 4:1, 7:1, 9:1, 100% hexamethyldisilazane). The sample was then evaporated in a chemical fume hood over 2 days. Prior to imaging the sample was gold spattered in a Leica CPD030. Cells in the colony periphery or invasion regions were imaged with the Photonic Bioimaging center at the University of Geneva using a JEOL JSM-6510LV system.

#### Macro photography

A tripod mounted Olympus E-M1 MkII camera equipped with a 60 mm F2.8 macro lens was used to take the images. *F. alba*-KpGe plates were illuminated continuously with a white led light (Thorlabs). To keep the open agar plates from drying, it was mounted in a plastic container over a water reservoir. The container and the camera lens were covered with a plastic wrap to create a closed humidity chamber. The time-lapse imaging was done using the camera’s in built time-lapse function. The time interval between images was 10 minutes.

#### Light microscopy

For liquid cultures, *F. alba* were inoculated into a 3 day-grown KpGe culture with a loop ∼24 hrs before imaging. Cells were allowed to settle onto a glass coverslip for 20 minutes then placed in an inhouse-designed metallic ring setup, which contains an o-ring seal that screws down to create a liquid chamber. Cells were then imaged. For agar surfaces, agar slabs of regions of interest were adhered to glass coverslips and inverted onto the metallic ring set up, which had an empty coverslip held by the o-ring. This created a hanging humidity chamber which prevented agar desiccation. For Figure 2A, the agar chunk was inverted directly on a glass coverslip, however, this results in cells rounding up and ultimate amoeba death, but preserves static structures while the imaging series was rapidly collected (<10 minutes after mounting).

Cells were imaged on an Olympus IX81 inverted wide-field epifluorescence microscope equipped with 20x/0.70 and 60x/1.25 phase objectives and the Orca-ER CCD camera (Hamamatsu). The IX81 microscope setup was controlled by Metamorph 7.5 (Molecular Devices, Sunnyvale, CA). Cell tickling was performed either on the Olympus IX81 by focussing a 488 nm on a ∼0.5 μm spot whose location was fixed in the field of view, or on an Olympus IX83 equipped with an iLas system which allowed us to easily control the laser spot position on the field of view. The Olympus IX83 was equipped with a 40x/0.6 Phase objective and a 488nm laser. VisiView software (Visitron Systems GmbH) controlled the Olympus IX83.

Confocal imaging of phalloidin fixed cells cells was done with the Photonic Bioimaging center at the University of Geneva using a Nicon Eclipse Ti1 microscope equipped with a CSU-W1 spinning disc (Yokogawa) using a 100X/NA 1.49 objective, a sCMOS 586 Prime 95B camera (Photometrics) and a 488 nm laser for illumination

#### Image analysis

All files used for the image analysis, and which are described below, can be downloaded from https://github.com/apicco/Fonticula_tracking.

#### Tracking of lobose and filose dynamics

We tracked the dynamics of Filose and Lobose single cells in liquid culture imaged with phase-contrast microscopy. In these images, cells appear slightly darker than the surrounding medium, and a bright halo surrounds them, making their thresholding hard. Therefore, we first processed the images so that the cells appear as bright and well-defined shapes that can be easily thresholded and thus tracked. To do so, we converted the images into 32-bit, then we inverted the LUT, and we performed a local background subtraction using a kernel radius of 35 pixels. The local background subtraction made cells stand out as bright shapes, while the LUT inversion made the bright halo look dark, enhancing the boundary of the cell shapes. The resulting images were ideal for thresholding and tracking.

Cell thresholding and tracking were performed in python, using the Scikit Image library (https://scikit-image.org/). The trajectories were collections of centres of mass positions of the shapes of the thresholded cells over time. We use the Trajalign library (http://apicco.github.io/trajectory_alignment/)^37^ to perform trajectory handling and analysis.

#### Tracking of single cells and collectives

We tracked single cells and collective cell dynamics by following their contractile vacuole dynamics, which appears dark in the brightfield images acquired with a 20x. We used Fiji/ImageJ to perform the tracking. In Fiji/ImageJ, we converted the images to 32-bit and added a small float (0.01). We then computed the log transform of the image. The small float prevented pixels from 0 intensity to diverge in the log transform. We then inverted the image by multiplication by -1. Now the dark vacuoles appear as bright spots. We background-subtracted the image with a rolling ball algorithm using a kernel radius of 9 pixels. Finally, we performed a Gaussian blur filter with kernel 2 to make vacuoles look more like spots to ease their recognition by the tracking algorithm. We tracked spot dynamics with Particle Tracker (Mosaic) using the following parameters: Radius = 7, Cutoff = 0, Percentile = 0.4, Linking range = 3, Displacement = 10.

#### Quantification of laser tickling of collectives

We classified the response of collectives to laser tickling in three categories: move backwards, keep moving forward, or undefined. The data used for the analysis are stored in Zenodo: https://doi.org/10.5281/zenodo.6303417.

The code and the quantification results are available on GitHub: https://github.com/apicco/Fonticula_collective_invasion.

### Quantification and statistical analysis

For statistical analysis, we used Python. Links to scripts used for quantifications are available in the key resources table. The number of data is listed in the text and or the figure legends. For hypothesis testing, we used the non-parametric Mann Whitney u test.

## Data Availability

All original code has been deposited on GitHub. Data that were too large to be hosted on GitHub were deposited at Zenodo. All Url and DOI are listed in the key resources table and the corresponding sections of the STAR Methods.

## Supplemental information

Document S1. Figures S1 and S2

Document S2. Article plus supplemental information
