# Supplementary material for: The cellular slime mold Fonticula alba forms a dynamic, multicellular collective while feeding on bacteria
Source: Curr Biol. 2022 May 9;32(9):1961–1973.e4. doi: 10.1016/j.cub.2022.03.018 (PMC9097593; doi:10.1016/j.cub.2022.03.018)
Supplement: Document S1. Figures S1 and S2 [file mmc1.pdf]

Current Biology, Volume *32*

Supplemental Information

The cellular slime mold *Fonticula alba* forms a dynamic, multicellular collective while feeding on bacteria

Christopher Toret, Andrea Picco, Micaela Boiero-Sanders, Alphee Michelot, and Marko Kaksonen

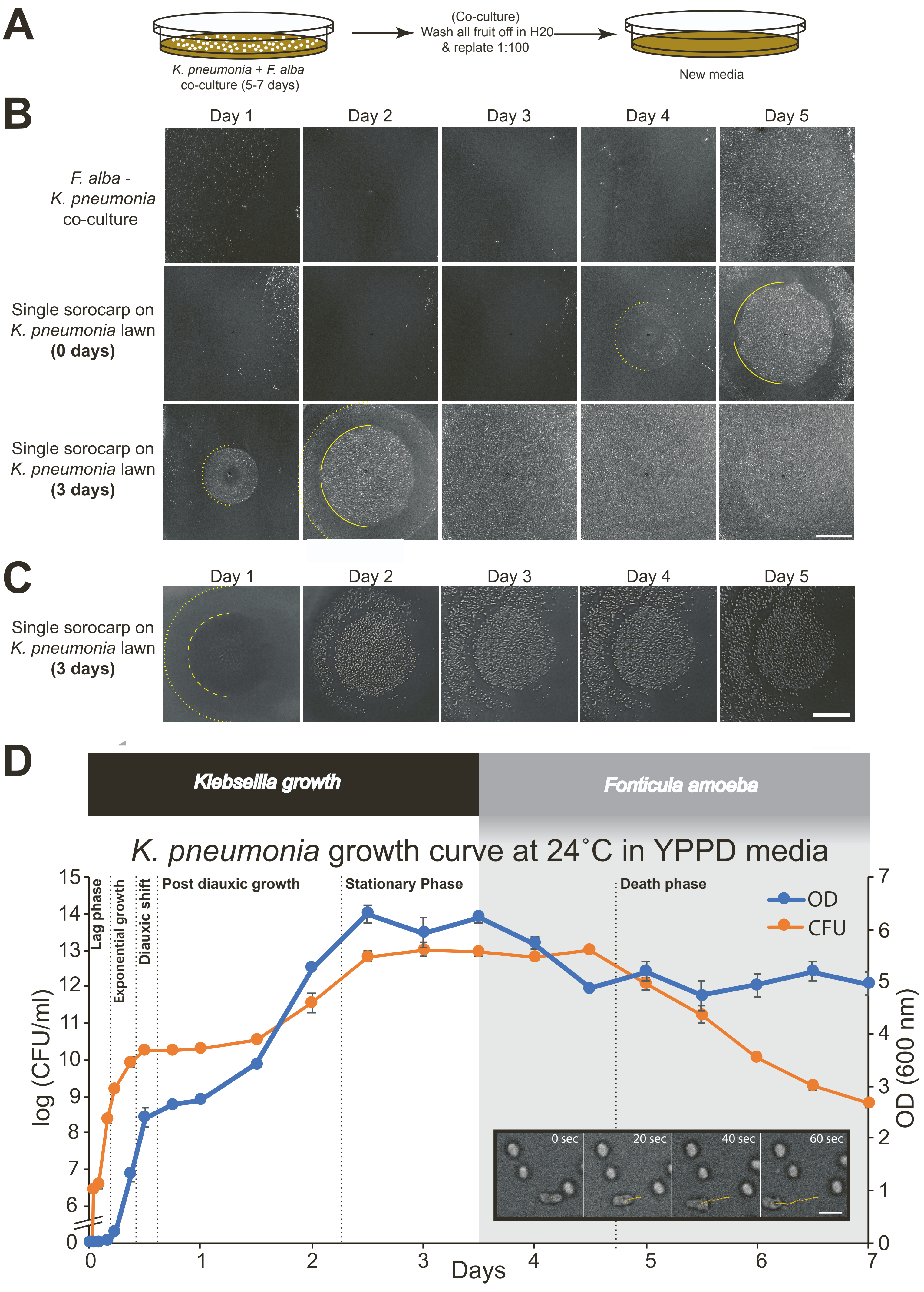
A
K. pneumonia + F. alba
co-culture (5-7 days)
(Co-culture)
Wash all fruit off in H20
& replate 1:100
New media
B
Day 1
Day 2
Day 3
Day 4
Day 5
F. alba -
K. pneumonia
co-culture
Single sorocarp on
K. pneumonia lawn
(0 days)
Single sorocarp on
K. pneumonia lawn
(3 days)
C
Day 1
Day 2
Day 3
Day 4
Day 5
Single sorocarp on
K. pneumonia lawn
(3 days)
D
Klebseilla growth
Fonticula amoeba
K. pneumonia growth curve at 24˚C in YPPD media
Lag phase
Exponential growth
Diauxic shift
Post diauxic growth
Stationary Phase
Death phase
OD
CFU
log (CFU/ml)
OD (600 nm)
Days
0 sec
20 sec
40 sec
60 sec

Figure S1. *F. alba* develops invasive growth on aged bacteria lawns, related to Figure 1. (A) Schematic of *K. pneumonia* - *F. alba* co-culutre method. (B) Images of the surface of an agar plate over time of indicated conditions. Solid line indicates fruit boundary. Dotted line indicates the invasive region. (C) Images of the surface of an agar plate over time of indicated conditions. Dashed line indicates bacteria depletion or feeding front. (D) Growth curve of *K. pneumonia* in YPPD media over 7 days plotted for Optical density (600 nm) and colony forming units (CFU). Growth phases are indicated on plot. Top bar indicates the time where bacteria and *F. alba* activities occur. Inset shows a brightfield montage example of a germinated amoeba among neighboring immotile spores detected 5 hrs after addition to a 6 day time point, with the amoeba mobility tracked in yellow over time. Scale bars = 1 cm (B and C) or 10 µm (D). See Figure 1.

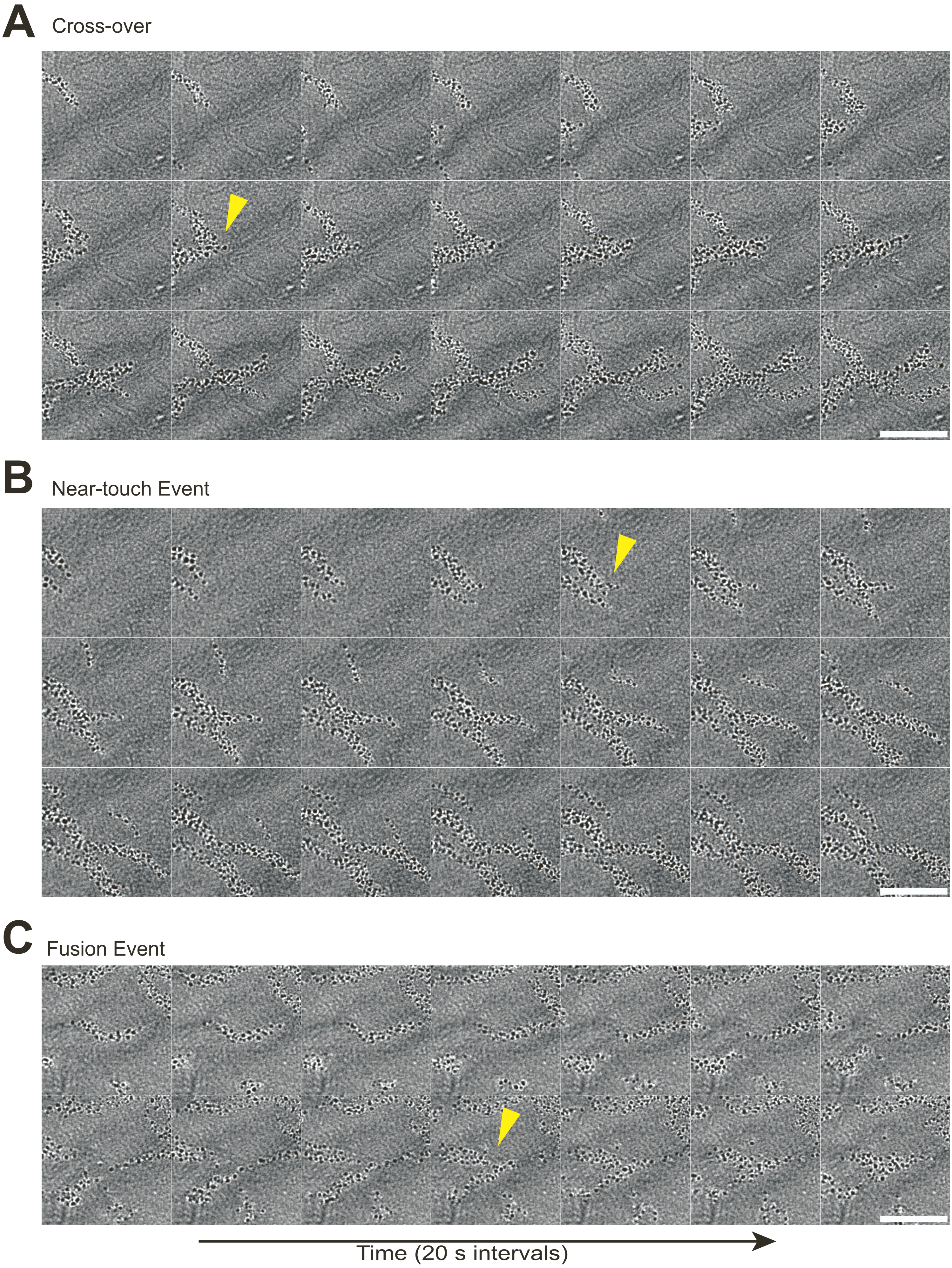
A
Cross-over
B
Near-touch Event
C
Fusion Event
Time (20 s intervals)

Figure S2. ***F. alba*** **invasive collectives are distinct**, **related to Figure 1.** 20 s montages cropped from Video S1. Yellow arrow identifies regions of invasion collective events. (A) Two collectives migrating in different directions meet and continue in the initial directions. (B) Two collectives migrating in close proximity and remaining distinct identities. (C) Two collectives merging into one collective. Scale bars = 100 µm. See Figure 1.
